# The physicochemical properties of microwave-assisted encapsulated anthocyanins from *Ipomoea batatas* as affected by different wall materials

**DOI:** 10.1002/fsn3.132

**Published:** 2015-01-03

**Authors:** Norazlina Mohd Nawi, Ida Idayu Muhamad, Aishah Mohd Marsin

**Affiliations:** 1 Bioprocess Engineering Department, Faculty of Chemical Engineering, Universiti Teknologi Malaysia Johor Bahru81310 Skudai, Johor, Malaysia; 2 IJN-UTM Cardiengineering Centre, V01 Universiti Teknologi Malaysia Johor Bahru81310 Skudai, Johor, Malaysia

**Keywords:** anthocyanins, encapsulating agent, maltodextrin, microwave-assisted, physicochemical properties, purple sweet potato

## Abstract

This study focuses on the impact of different wall materials on the physicochemical properties of microwave-assisted encapsulated anthocyanins from *Ipomoea batatas*. Using the powder characterization technique, purple sweet potato anthocyanin (PSPAs) powders were analysed for moisture content, water activity, dissolution time, hygroscopicity, color and morphology. PSPAs were produced using different wall materials: maltodextrin (MD), gum arabic (GA) and a combination of gum arabic and maltodextrin (GA + MD) at a 1:1 ratio. Each of the wall materials was homogenized to the core material at a core/wall material ratio of 5 and were microencapsulated by microwave-assisted drying at 1100 W. Results indicated that encapsulated powder with the GA and MD combination presented better quality of powder with the lowest value of moisture content and water activity. With respect to morphology, the microcapsule encapsulated with GA + MD showed several dents in coating surrounding its core material, whereas other encapsulated powders showed small or slight dents entrapped onto the bioactive compound. Colorimetric analysis showed changes in values of *L*, a*, b*, hue and chroma in the reconstituted powder compared to the initial powder.

## Introduction

The natural colorant of purple sweet potatoes (*Ipomoea batatas*) has been developed in Japan, Korea, New Zealand and other countries to fulfil a rapidly growing demand in the health food market (Steed and Truong 2008). Purple sweet potatoes have intense purple color due to the accumulation of anthocyanins which could be extracted using distilled water or ethanol or other solvent (Terahara et al. 2004; Ahmed et al. 2010). Purple sweet potato anthocyanins (PSPAs) are good sources of mono- or di-acylated forms of cyanidin and penionidin, which contribute to high antioxidant activity as compared to white, yellow and orange colored sweet potatoes (Teow et al. 2007; Steed and Truong 2008; Sushma et al. 2012). Research into the properties of purple sweet potato indicated that extracted anthocyanins are good for health since they have numerous beneficial effects such as anticarcinogen, antimutagenicity, antihypertensive and scavenging free radical (Ahmed et al. 2010).

Anthocyanins are easily dissolved in water and are responsible for fruit and flower coloration by themselves (Zhang et al. 2014). This natural colorant has widely replaced synthetic colorants, but had encountered color stability problems when exposed to environment during processing and storage conditions including temperature, pH, oxygen, enzymes, light, free radical (Ahmed et al. 2010). Therefore, the food industry is continuously looking to encapsulation technology as a novel and inexpensive method aimed at improving the pigment stability of bioactive compounds and to increase the shelf-life. Encapsulation is a technique by which small particles of solid, liquid or gas are coated or entrapped by a coating material to form a microcapsule. Encapsulation also protects sensitive food components against degradation reactions and loss of volatility (Charikleia and Constantina 2014). The droplets or particles are called active or core material while the coating material is also known as shell, wall material, matrix, carrier or encapsulant (Madene et al. 2006). In this study, microwave-assisted encapsulation can be an alternative technique for producing encapsulated PSPAs.

Microwave-assisted encapsulation is a new and economical method for preservation of natural colorants by entrapping the core material with the coating agent. It promotes good quality, easy handling, low water activity and extended shelf life of ingredients (Desai and Park 2005). Microwave heating transfers energy via two mechanism: dipole rotation and ionic conduction which displace charged ions present in the solute via the solvent (Winny and Valéria 2012). Mandal et al. (2007) reported that microwave-assisted encapsulation is very useful to shorten the extraction time by using the microwave energy to heat the solvent and discharge the contents of the plant materials into the liquid phase.

Due to problems like stickiness and hygroscopicity in producing microwave-assisted powders, addition of some encapsulating agents could be useful. The most commonly used encapsulating agents are maltodextrin and gum arabic. Some studies used these encapsulating agents for drying of fennel oleoresin (Charikleia and Constantina 2013, 2014) and phenolic antioxidants (Pitchaon et al. 2013), resulting in powders with low moisture content and *a*_w_ values, as well as better solubility, lower hygroscopicity and higher vitamin C retention. Maltodextrins are oligosaccharides produced by starch analysis and have less than 20 dextrose equivalents (DE) which determines the degree of starch polymer hydrolysis and average molecular weight (Langrish et al. 2007). Clearly, they have the ability to form matrices which are essential in forming wall systems. In selecting the wall material, maltodextrin is very useful because of good balance between cost and effectiveness since it has low viscosity at a high solid ratio and is available in different average molecular weights (Apintanapong and Noomhorm 2003; Madene et al. 2006). Utilizing maltodextrin as an encapsulating agent has given good results in minimizing wall deposition. Bhandari et al. (1993) found that a certain amount of maltodextrin had to be added to prevent an excessive amount of product from sticking to the walls of the spray dryer. They also found that more wall deposition occurred due to an increase in stickiness during drying, when the ratios of fruit juice to maltodextrin increased (Langrish et al. 2007).

Gum arabic is a natural product of *Acacia*, consisting of d-glucuronic acid, l-rhamnose, d-galactose and l-arabinose with approximately 2% protein (Dickinson 2003; Gharsallaoui et al. 2007). Krishnan et al. (2005) found that gum arabic was a better wall material for encapsulation of cardamom oleoresin compared to maltodextrin and modified starch and the microcapsules revealed free flowing character. Gum arabic is mostly gum used in the encapsulation process because of its solubility, low viscosity, emulsification characteristics and good retention of volatile compounds. Moreover, this gum is ideally suited to the encapsulation of lipid droplets as it fulfills the roles of both surface-active agent and drying matrix, thus preventing the loss of volatiles exposed to the atmosphere. However, its application in the industry is very restricted because of high cost, limited supply and quality variation (Madene et al. 2006). Producing powders using different wall materials resulted in different physicochemical properties such as water activity, moisture content, shelf life and hygroscopicity, depending on the structure and characteristics of each wall material.

There is very limited scientific study concerning the encapsulation of PSPAs using microwave-assisted encapsulation. Based on the above reasons, this study was conducted to investigate the feasibility of microwave-assisted encapsulation of PSPA extracts and to evaluate the use of different wall materials on the physicochemical properties of encapsulated PSPA powders with respect to moisture content, water activity, dissolution, color hygroscopicity and microcapsule morphology.

Currently, there is high interest among food researchers in the use of microwave processing due to its high potential capability. Microwave drying is an innovative and new method where shells which have different dielectric constant can fuse and cover the core material (Abbasi and Rahimi 2008). Microwave drying also has a potential to serve as a rapid encapsulation technique that can be applied in the food industry. The advantages of using microwave drying include shorter drying time, low cost, improved product quality and flexibility in producing a variety of dried products (Hangi and Amanifard 2008).

The aim of this study was to identify the optimum wall material on the physicochemical properties of microwave-assisted encapsulated anthocyanins from *I. batatas*. The PSPA powders were analysed for moisture content, water activity, dissolution time, hygroscopicity, color and morphology.

## Materials and Methods

### Sample preparation

Fresh purple sweet potatoes were purchased at the local market and washed in running tap water to eliminate extraneous matter. The potatoes were refrigerated from 5°C to about 10°C before extraction to help control sprout growth, reduce senescent sweetening and minimize spoilage duration. The potatoes were cut into pieces approximately 0.2 cm for extraction.

### Preparation of PSPA extract

The potato slices were steamed and mashed with water–95% ethanol (ratio 4:1, v/v) at a ratio of 1:3 for 10 min. After cooling to room temperature, the extract was filtered using cheesecloth. The filtered extracts were centrifuged at 10,733 *g* for 10 min to remove precipitants. The supernatant was concentrated to 6% solid content by using a rotary evaporator at 70°C. The extracts were kept frozen at −20°C until further analysis.

Analysis of PSPA extract was carried out to determine the pH, color, total solid and total anthocyanin content. Total soluble solid of PSP extract was determined using an Atago Refractometer (Atago, Tokyo, Japan) at room temperature. pH was measured using a pH meter. Color parameters (*L*, a*, b*, *C* and H) were measured using a color meter (Konica Minolta, Shanghai, China).

### Total anthocyanin content

Total anthocyanin content was determined using the spectrophotometric pH-differential method described by Wang and Xu (2007) using two buffer systems: potassium chloride buffer, pH 1.0 (0.025 mol/L) (125 mL of 0.2 mol/L KCl and 375 of 0.2 mol/L HCl) and sodium acetate buffer, pH 4.5 (0.4 mol/L) (400 mL of 1 mol/L sodium acetate, 240 mL of 1 mol/L HCl and 360 mL of water). The absorbance was measured at 520 and 700 nm with distilled water as blank using a UV–VIS spectrophotometer. The absorbance difference between the pH 1.0 and pH 4.5 samples was calculated:

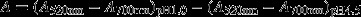
1

The total anthocyanin content was calculated as cyanidin-3-glucoside according to the following equation:

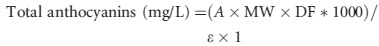
2where MW (molecular weight) = 449.2 g/mol for cyanidin-3-glucoside; DF is the dilution factor; 1 = pathlength in cm; *ε *= 26,900 molar extinction coefficient in L/mol/cm for cyanidin-3-glucoside; 1000 = conversion from g to mg. All analysis were performed in triplicate (*n *=* *3).

### Preparation of microcapsules using microwave-assisted encapsulation

Wall materials (GA, maltodextrin, GA: maltodextrin, 1:1) were combined with pigment concentrate (6% solid content) and stirred to homogeneity with a mixer for 30 min. Each of the wall materials was added until 20% of the final solid content was reaching. The mixture was placed inside round glass plates and placed in a domestic microwave oven. Each mixture was treated for up to 600 sec (depending on the type of wall material) at microwave power intensities (1100 W).

### Analysis of microcapsule powder

#### Water activity

The water activity of the samples was determined using a portable water activity analysis set (Water activity; Hygrolab, Hauppauge, NY). Duplicates samples were measured at 25°C.

#### Dissolution test

A sample of 50 mg of dried powder was mixed with 1 mL distilled water in a test tube. The solution was mixed in a vortex (ASASI Vortex Mixer WM-20; Tech-Lab Scientific, Selangor, Malaysia) at moderate speed. The time required for the powder to dissolve in water was recorded. The test was carried out in triplicate.

#### Color evaluation

L*, a*, b* color values of the samples were measured using a Minolta CT-310 Colorimeter (Konica Minolta). C* for the metric chroma and *H*° for the hue angle were calculated by the transformation of a* and b* the following equations:

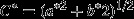


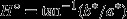


On the chromatic circle, *H*° values are stepped from 0° to 360° (magenta red) across a continuously fading hue circle, the other reference values of which are 90° (yellow), 180° (bluish green) and 270° (blue).

#### Scanning electron microscopy

Particle structures of the powder microcapsules were evaluated by a scanning electron microscope (SEM) (Philips XL 40, Holland, The Netherlands). Powders were attached to SEM stubs using a two-sided adhesive tape. Samples were coated with 200 Å gold under vacuum before examination. SEM was operated at 20 kV × 250.

## Results and Discussion

### Physicochemical analysis of PSPA extract

Table1 shows the physicochemical characteristics of purple sweet potato extract using distilled water added with ethanol (40%) at a ratio of 1:3. It was found that the purple sweet potato extract has 9.9 ± 1.13 °Brix. It can be seen that the pH of PSP extract was as high as 6.34, which implied that it is susceptible to microbial growth (Quek et al. 2007). The color values of purple sweet potato were measured as L*, a*, b* values. The chroma and hue were calculated as 4.88 ± 0.31 and 79.69 ± 7.09 respectively. Color measurement is very essential as a quality indicator since it promotes sensory attractiveness and quality of the powders.

**Table 1:** Physical characteristics of purple sweet potato extract.

Parameter	Value
Total soluble solid (°Brix)	9.9 ± 1.13
pH	6.34 ± 0.38
Total anthocyanin content (mg/L)	50.50 ± 3.21
Color parameters	
L^*^	31.28 ± 0.12
a^*^	0.93 ± 0.71
b^*^	4.68 ± 0.07
C^*^	4.88 ± 0.31
*H*°	79.69 ± 7.09

### Effect of encapsulating agent

**Figure 1:**
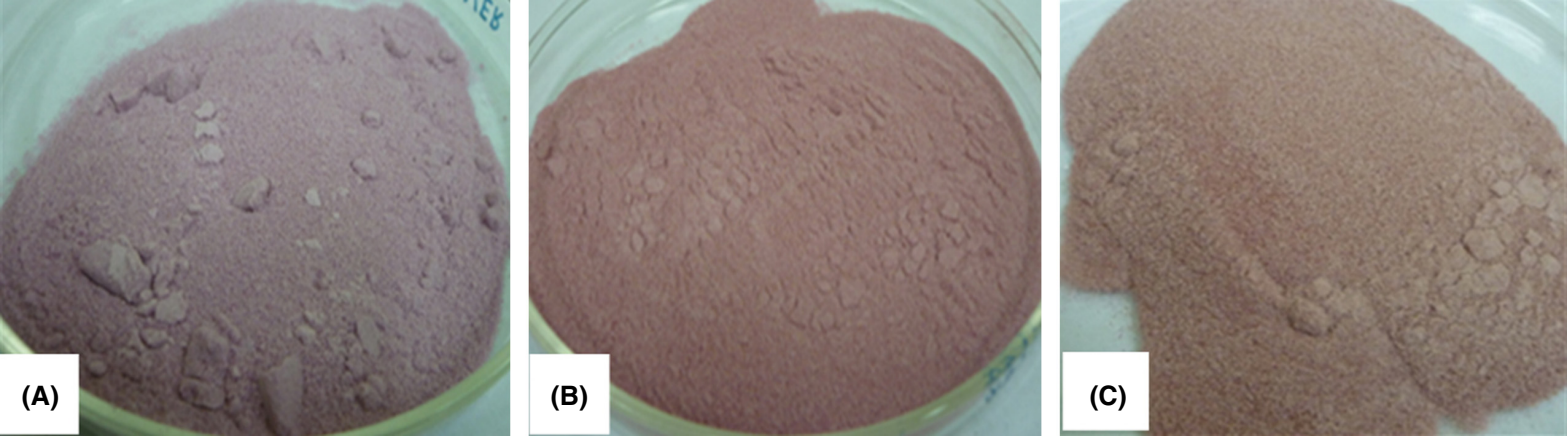
Encapsulated anthocyanins of *Ipomoea batatas* powder using microwave assisted using different wall materials: (A) MD (maltodextrin); (B) GA + MD (gum arabic + maltodextrin); (C) GA (gum arabic).

To be a good wall material, the most critical properties were its capability to hold and entrap all core material within its structure during processing and storage. If the wall material did not reach these properties, unnecessary loss of core material during the processing will occur (Dian et al. 1996). From the observations, adding encapsulating agent to the extract could easily produce the powder. Without addition of any encapsulating agents, there was hardly any powder collected. Therefore, three types of encapsulating agents (Fig.1) were added to microwave-assisted powders, which are maltodextrin, gum arabic and combination of gum arabic and maltodextrin (50:50). This was done to investigate the physical properties of different encapsulated powders. Maltodextrin was used because it possessed better nutrient binding properties (Cai and Corke 2000). Maltodextrin was confirmed to be a good encapsulating agent because it could alter the surface stickiness of low molecular weight sugars and organic acids, thereby facilitating drying and reducing the stickiness of the powder (Endut and Osman 2009). Studies by Duangmal et al. (2008) proved that the addition of maltodextrin and trehalose retarded anthocyanin degradation in freeze-dried powder. Also, Quek et al. (2007) found that the addition of maltodextrin could aid the drying process in spray drying of watermelon juice, thus improving the yield of the product.

### Moisture content and hygroscopicity

Table2 shows the value of moisture content and hydroscopic moisture of encapsulated anthocyanin powder with different wall materials. Moisture content represents the water composition in a food system. According to Dian et al. (1996), moisture content played an important role in microencapsulated oil because higher moisture content emerged as a major factor in fat oxidation and thus affected flowability of powders. From our study, the powder produced with gum arabic showed the highest value of moisture contents while particles from a combination of gum arabic and maltodextrin indicated the lowest value of moisture content. This result is in agreement with those of Tonon et al. (2009), which indicated that gum arabic had the highest moisture content compared to other encapsulating agents. The addition of different wall materials to the extract affected various moisture content values. According to Tonon et al. (2009), moisture content was affected by the chemical structure of gum arabic and maltodextrin, which have a high number of hydrophilic groups, and thus can easily bind to water molecules from the ambient air during powder handling. A study on dried watermelon powder by Quek et al. (2007) showed that an increasing percentage of maltodextrins exhibited decreasing moisture content of the powder. By adding maltodextrin to the feed, the total solid content would be increased, thus reducing the amount of water for evaporation. However, if the amount of maltodextrin were too high, it would lessen the quality of powder because the nutrient of watermelon juice would be diluted. Higher initial water content of liquid resulted in higher moisture content of the final product (Thankitsunthorn et al. 2009).

**Table 2:** Moisture content and hygroscopicity of encapsulated anthocyanin of *Ipomoea batatas* powder.

Sample	Moisture content (%)	Hygroscopic moisture (g/100 g) (%)
Maltodextrin	10.2281 ± 0.04a	10.028 ± 2.11a
Gum arabic	15.5753 ± 1.90b	18.736 ± 0.15b
Maltodextrin + gum arabic	8.7878 ± 1.27c	15.181 ± 0.39c

Mean ± SD from triplicate determination. Means within a column with different letters are significantly different (*P* < 0.05).

Hygroscopicity plays an important role in determining the shelf life and storage of products. According to Al-Kahtani and Hassan (1990), hygroscopicity was one of the major problems involved in determining the stability of spray-dried roselle powder. As shown in Table2, the addition of maltodextrin, gum arabic and combination of gum arabic and maltodextrin as wall material significantly affected the hygroscopicity of the powder. From our study, the sample produced with maltodextrin was the least hygroscopic. The highest hygroscopicity of the powders was exhibited by gum arabic microcapsules with 18.736% followed by the combination of maltodextrin and gum arabic (15.181%). The powder encapsulated with maltodextrins showed the least hygroscopic moisture with 10.028%. The number of hydrophilic groups present in the structure of each wall material contributed to the variation of water adsorption. The hygroscopicity value corresponded to the moisture content, which meant that the higher the moisture content, the higher the hygroscopic value. The product showed better stability with adding maltodextrin as stabilizer because it could reduce reactant mobility. Moreover, it assisted the powder in changing to “sorption gel” (Chronakis 1998; Duangmal et al. 2008).

Tonon et al. (2009) revealed that the samples produced with maltodextrin 20DE and with gum arabic were more hygroscopic than maltodextrin 10DE and tapioca starch. They related that both gum arabic and maltodextrin 20DE had higher effects on hydrophilic groups and hence easily adsorb moisture from the surrounding air. In contrast, the maltodextrin 10DE and tapioca starch have fewer hydrophilic groups and consequently exhibited less water adsorption than others.

Powder hygroscopicity was also affected by the concentration of wall material. It is approved by Tonon et al. (2008), working on the physicochemical properties of açai powder produced by spray drying using maltodextrin. The highest concentration of maltodextrin used obtained lowest hygroscopicity values. This is because maltodextrin is a material with low hygroscopicity, confirming its efficiency as a carrier agent. Similar results were obtained by Cai and Corke (2000) and Rodríguez-Hernández et al. (2005), working with spray drying of betacyanin pigments and cactus pear juice, respectively, both using maltodextrin which verified that increasing maltodextrin concentrations cause reduction in hygroscopicity. The same results were reported by Endut and Osman (2009), who revealed that powder hygroscopicity would be decreased with increased maltodextrin percentage.

### Water activity

**Figure 2:**
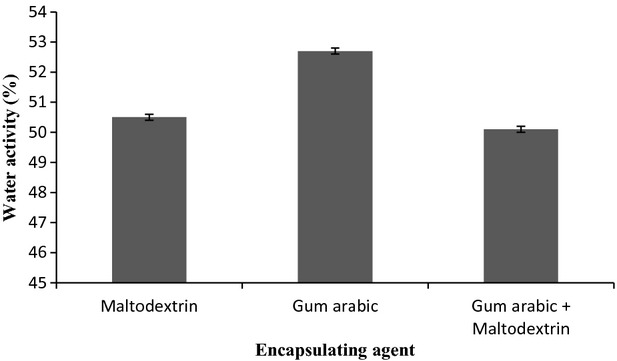
Graph of water activity using different encapsulating agent.

Water activity is a very essential index for microwave-assisted powders as it determines the shelf life of the product. Water activity is defined as the ratio of vapour pressure of water in a food system to vapour pressure of pure water at the same temperature. Water activity varies with moisture content as it measures the availability of free water in a food system which is responsible for any biochemical reactions, whereas the moisture content represents the composition of water in a food system (Quek et al. 2007). From Fig.2, the range of water activity of the powders produced from *I. batatas* is very slight, between 0.5 and 0.52. This range is considered microbiologically stable. Powders produced with the combination gum arabic and maltodextrin showed the lowest water activity, followed by those produced with maltodextrin alone. Particles produced with gum arabic showed the highest water activity. Such results are similar and in agreement with those of moisture content and the differences on *a*_w_ values can also be related to the chemical structure of these agents. Higher water activity indicates that there is more free water available for microorganism growth and biochemical reactions and thus shorter product shelf life (Tonon et al. 2009). Basically, food with *a*_w_ less than 0.6 is microbiologically stable (Endut and Osman 2009). A similar range of *a*_w_ values were obtained by Quek et al. (2007) for spray-dried watermelon.

### Dissolution test

**Figure 3:**
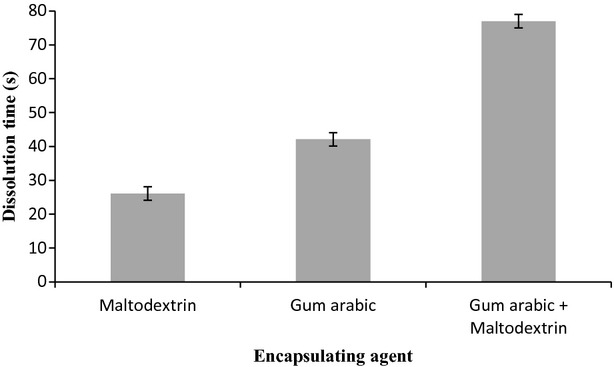
Graph of dissolution test using different encapsulating agent.

The dissolution test measures the time required by the powder to fully reconstitute in water by using vortex. The results are shown in Fig.3. Powder encapsulated with a combination of gum arabic and maltodextrin gave higher dissolution time, followed by particles produced with gum arabic. Particles produced with maltodextrin alone showed the lowest dissolution time, which means that the time taken for the powder to dissolve in water is relatively shorter compared to others. From the results, it can be mentioned that maltodextrin is highly soluble compared to gum arabic.

The time taken from the dissolution test can be related to the moisture content of the final product. From Table1 and Fig.3, the lower the moisture content of the powder, the more difficult it is for the powder to reconstitute in water. Higher moisture content of the powder resulted in higher tendency of the powder to agglomerate, which helped to increase the reconstitution of the powder (Endut and Osman 2009). Similar results were obtained by Quek et al. (2007) working with encapsulation of spray-dried watermelon powders using two different concentrations of maltodextrin (3% and 5%). The authors verified that there was a positive relationship between dissolution and moisture content of the powders. It meant that when the moisture content of powder increased, the time of dissolution decreased.

### Color measurement

Color is represented by L*, a* and b* where L* measures the lightness of the sample (from black to white), a* measures the redness and greenness, b* measures the yellowness and blueness. Hue angle measures the property of the color, and was calculated by the formula *H*° = tan^−1^(b*/a*). The hue angle values vary from 0° [(pure red), 90° (pure yellow), 180° (pure green)] to 270° (pure blue). Chroma indicates the color intensity or saturation, and was calculated by the formula (a*^2^ + b*^2^)^1/2^ (Kha et al. 2010). Results of the color measurement for encapsulated powders and the reconstituted powder are shown in Table3.

**Table 3:** Colorimetric results of microwave-assisted powder and reconstituted powder at different wall materials.

Sample	L^*^	a^*^	b^*^	Chroma	Hue
Dry powder					
MD	52.65 ± 1.39a	13.97 ± 1.37a	1.2 ± 0.24a	14.02 ± 1.34a	5.0 ± 1.47a
GA	44.98 ± 0.64b	11.25 ± 0.97a	5.11 ± 0.12b	12.37 ± 0.83a	24.56 ± 2.29b
GA + MD	48.85 ± 0.68c	10.02 ± 1.96a	3.03 ± 0.05c	10.48 ± 1.88a	17.37 ± 3.28b
Reconstituted powder					
MD	31.82 ± 4.60a	2.82 ± 0.40a	3.93 ± 0.47a	4.86 ± 0.15a	54.28 ± 7.09a
GA	34.48 ± 0.21a	2.17 ± 0.19b	0.17 ± 0.00a	2.18 ± 0.19a	4.41 ± 0.38b
GA + MD	33.28 ± 0.40a	1.95 ± 0.45b	0.42 ± 0.07a	1.99 ± 0.45a	12.14 ± 0.72b

MD, maltodextrin; GA, gum arabic; GA + MD, combination of gum arabic and maltodextrin.

Means ± SD, *n* = 3. Means within a column with different letters are significantly different (*P* < 0.05).

It was found that the lightness for all encapsulated powders had a significant effect (*P* < 0.05) on each other. Encapsulated powders with MD (*P* < 0.05) showed the highest lightness value, followed by GA + MD and GA. However, in the reconstituted powder, encapsulated with GA, MD and GA + MD showed an insignificant difference (*P* > 0.05) and slight difference between each other. The a* value showed reddish color in powder and reconstituted powder, indicating that encapsulated anthocyanins with MD exhibited higher reddish color followed by GA and GA + MD.

Table3 shows that the chromaticity for both powder and reconstituted powder forms showed insignificant difference (*P* > 0.05). The highest chroma in both dry powder and reconstituted powder was that encapsulated with MD followed by GA and GA + MD. Color measurement is an important quality indicator since it reflects sensory attractiveness and quality of the powders produced during the microwave drying process.

### Particle size and microstructure

**Figure 4:**
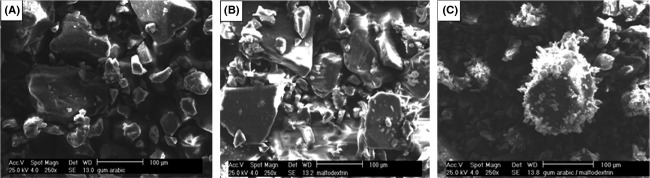
Micrographs of microcapsules microwave dried powders of purple sweet potato anthocyanins pigment produced with different wall material A) gum arabic, B) maltodextrin, C) gum arabic + maltodextrin.

The analysis of the surface of PSPA powder particles were carried out using SEM. Figure4 shows the SEM micrographs of the powders produced with different encapsulating agents. The resulting powders had a particle size of approximately 100 *μ*m. It was found that all particles produced had a smooth surface and flake-like structure, which is typical of microcapsules prepared by microwave drying. SEM micrographs of GA + MD (Fig.4C) showed more dents surrounding the core material, which means that this combination of wall materials had higher efficiency encapsulation, whereas few dents appeared in micrographs of encapsulated maltodextrin and gum arabic alone.

## Conclusion

Microcapsule powder was produced using microwave-assisted powders with different wall materials. Addition of wall material could reduce the stickiness of the products and modify the physicochemical properties of microwave-assisted powders. The results showed that the addition of the combination gum arabic and maltodextrin as encapsulating agent to the PSPA extract produced better quality microcapsules. This was approved by the results which indicated that moisture content and water activity were the lowest values while dissolution time was longer compared to other encapsulated powders. Lightness, chromaticity and hue showed a decreasing trend in the reconstituted powder compared to the powder form. In order to evaluate better the stability of powders, the storage time should extent more than 2 month. The physicochemical properties of food powders are very important for ensuring the production of high-quality purple sweet potato powders.
